# P-953. Stratifying Reaction Histories of Hospitalized Patients with a Beta-Lactam Allergy to Improve Beta-Lactam Utilization in a Large Healthcare System

**DOI:** 10.1093/ofid/ofaf695.1155

**Published:** 2026-01-11

**Authors:** Aaron Oliver, Emma Ku, Erin Weslander, Rishita Shah, Jaime Borkowski, Michael Postelnick, Sarah Sutton, Michael Dickens, Michael Carey, Stephanie Chang, Radhika S Polisetty, Sheila K Wang

**Affiliations:** Northwestern Medicine, Chicago, IL; Midwestern University College of Pharmacy, Downers Grove, Illinois; Northwestern Memorial Hospital, Chicago, IL; Northwestern Lake Forest Hospital, Lake Forest, IL, Illinois; NM Delnor Hospital, Geneva, Illinois; Northwestern Medicine, Chicago, IL; Northwestern University, Chicago, Illinois; Northwestern Medicine, Chicago, IL; Northwestern Medicine, Chicago, IL; Northwestern Medicine - Huntley Hospital, Huntley, Illinois; Midwestern University College of Pharmacy/ Northwestern Medicine Central DuPage Hospital, Winfield, Illinois; Midwestern University College of Pharmacy/Northwestern Memorial Hosptial, Downers Grove, Illinois

## Abstract

**Background:**

A beta-lactam allergy label (BAL) limits healthcare providers' options for antibiotics, forcing them to use non-beta-lactam alternatives. The inappropriate use of these alternatives can lead to collateral complications and poor clinical outcomes. In the U.S., 34 million people (10%) are reported to have a BAL; however, over 95% are incorrectly labeled and do not have a true allergy. True beta-lactam allergies can vary in severity, timing, and type of reaction. This study aimed to risk-stratify the reaction histories of hospitalized patients with a BAL across a large healthcare system to identify antimicrobial stewardship strategies for improving the use of beta-lactams.

**Methods:**

A retrospective review was conducted on adult patients hospitalized with a BAL who stayed overnight at one of seven Northwestern Medicine (NM) hospitals between August 1, 2020, and August 1, 2022. The patients were categorized based on their history of an allergic reaction into five risk levels: negligible risk, low risk, moderate risk, and high risk/avoid. The findings from this review were used to develop targeted antimicrobial stewardship interventions aimed at improving the use of beta-lactams across the seven hospitals within the NM system.

**Results:**

A total of 25,768 patients (13.1%) had a BAL with 13,957 patients qualifying for inclusion in the study. Risk stratification using the NM Beta-Lactam Allergy Risk Assessment and Clinical Pathway for Non-Critically Ill Patients (figure 1) identified 3,768 patients (27%) at negligible risk, 6,916 (50%) at low risk, 2,575 (18%) at moderate risk, and 698 (5%) at high risk/avoid. Over 75% of patients with BAL had allergic reactions classified as negligible to low risk. As a result, a system-wide Beta-Lactam Drug Challenge Guidance Document (figure 2) was developed to target low-risk patients for direct or step-wise beta-lactam challenges to improve the usage of beta-lactam antibiotics.

**Conclusion:**

The prevalence of a BAL among NM hospitals is 13.1%, which aligns with existing literature. Classifying reaction histories to beta-lactam antibiotics using pre-defined risk categories can assist in developing antimicrobial stewardship strategies. This approach aims to improve the appropriate use of beta-lactams across a large academic healthcare system.

**Disclosures:**

All Authors: No reported disclosures

